# Intra-Tumour Genetic Heterogeneity and Prognosis in High-Risk Neuroblastoma

**DOI:** 10.3390/cancers13205173

**Published:** 2021-10-15

**Authors:** Amparo López-Carrasco, Ana P. Berbegall, Susana Martín-Vañó, Maite Blanquer-Maceiras, Victoria Castel, Samuel Navarro, Rosa Noguera

**Affiliations:** 1 Department of Pathology, Medical School, University of Valencia-INCLIVA, 46010 Valencia, Spain; amparolopezcarrasco@gmail.com (A.L.-C.); ana.berbegall@uv.es (A.P.B.); susana.martin@uv.es (S.M.-V.); maiteblanquer@gmail.com (M.B.-M.); Samuel.Navarro@uv.es (S.N.); 2 CIBER of Cancer (CIBERONC), 28029 Madrid, Spain; 3 Clinical and Translational Oncology Research Group, Investigation Institute La Fe, 46026 Valencia, Spain; castel_vic@gva.es

**Keywords:** SNPa, ctDNA, segmental chromosomal aberration, genomics, *MYCN* amplification, tumour microenvironment

## Abstract

**Simple Summary:**

Neuroblastoma (NB) is the most common extra-cranial solid paediatric cancer and is responsible for 15% of childhood cancer deaths. Patients with NB are characterized by presenting a very heterogeneous clinic (inter-tumoural heterogeneity) and also both spatial and temporal intra-tumour heterogeneity (ITH) reflected in their genetic aberrations, which may be the consequence of the coexistence of different microenvironments within the tumour. Applying pangenomic techniques to detect genomic aberrations in different biopsies (solid and liquid) of high risk NB (HR-NB) we have detected spatial ITH in a surprisingly high percentage (almost 40%) of the studied cohort. Moreover, a positive association between this heterogeneity and survival has been found. Confirming these results, combining tumour material analysis in a large cohort of HR-NB will have a major impact in the genetic diagnosis routine procedure, and would also entail a revision of the prognosis of patients with ITH.

**Abstract:**

Spatial ITH is defined by genomic and biological variations within a tumour acquired by tumour cell evolution under diverse microenvironments, and its role in NB patient prognosis is understudied. In this work, we applied pangenomic techniques to detect chromosomal aberrations in at least two different areas of each tumour and/or in simultaneously obtained solid and liquid biopsies, detecting ITH in the genomic profile of almost 40% of HR-NB. ITH was better detected when comparing one or more tumour pieces and liquid biopsy (50%) than between different tumour pieces (21%). Interestingly, we found that patients with ITH analysed by pangenomic techniques had a significantly better survival rate that those with non-heterogeneous tumours, especially in cases without *MYCN* amplification. Moreover, all patients in the studied cohort with high ITH (defined as 50% or more genomic aberration differences between areas of a tumour or simultaneously obtained samples) survived after 48 months. These results clearly support analysing at least two solid tumour areas (separately or mixed) and liquid samples to provide more accurate genomic diagnosis, prognosis and therapy options in HR-NB.

## 1. Introduction

In addition to genetic and clinical inter-tumour heterogeneity, which refers to variations found between different patients in tumours and/or behaviour, tumours can also present with spatial and/or temporal intra-tumour heterogeneity (ITH). Spatial genomic ITH is characterised by genomic variations within a tumour lesion, or between different tumours located in the patient at the same time point in the case of metastatic disease, and consists of multiple cells or sub-clones, each possessing different genomic profiles [1]. Temporal genomic ITH denotes genetic variation over the course of disease progression and has been observed mainly when comparing treatment-naïve and matched relapsed tumours [2]. Both ITH types can be a result of clonal evolution due to natural selection among tumour cells, or in the absence of strong selection can also emerge through the mechanism of genetic drift [2,3]. This diversity most likely arises as a way for the tumour to adapt to changing microenvironmental conditions and/or as a tool for altering its malignant potential [4].

In neuroblastoma (NB), ITH has been associated with a spectrum from favourable to unfavourable patient outcomes [5,6]. NB is the most common extra-cranial solid paediatric cancer and is responsible for 15% of childhood cancer deaths [7]. The International NB Risk Group (INRG) [8] has defined a currently-used prognosis classification according to clinical and biological parameters. Patients classified as high risk NB (HR-NB) have the poorest outcomes, with <50% survival [9]. Adverse prognostic factors include metastatic stage, age ≥18 months at diagnosis, undifferentiated or poorly differentiated histopathology and specific genetic abnormalities, such as *MYCN* amplification (MNA) and segmental chromosomal aberrations (SCAs) [10]. Recent molecular classifications have added new markers such as *ALK, TERT, ATRX, TP53* and alternative lengthening of telomeres (ALT) abnormalities, to typify around 80% of HR-NB [11]. Tumour microenvironment (TME) and particularly extracellular matrix (ECM) features are also emerging as important factors contributing to tumour prognosis and heterogeneity [12,13]. In this regard, our group has previously described a common aggressive pattern of rigid ECM in HR-NB [14], rich in cross-linked collagen III fibres, poor in glycosaminoglycans, supporting sinusoidal vascular structures (blood and lymphatic) and with a high amount of territorial vitronectin (located in cytoplasmic compartments and a thin layer around tumour cells) [15,16,17,18]. In a recent study we observed the dramatic impact of ECM stiffness on ITH and clonal evolution of a MNA NB cell line, in which dominant clones were selected when cultured in stiff 3D-bioprinted hydrogels and in xenograft tumours; these changes were not found in an *ALK* mutated NB cell-line [3,19]. A continuous interplay therefore exists between TME and genetics, tumour cell proliferation, aggressiveness and migration [20].

In circulating tumour DNA (ctDNA) studies, liquid biopsy is viewed as an emerging tool for identifying genetic alterations across the whole tumour, and is particularly significant in highly heterogeneous neoplasia. Generally, HR-NB has been shown to release large quantities of ctDNA [21]. In fact, recent studies have demonstrated broad ITH when comparing ctDNA with DNA from NB tissue using high-throughput techniques, suggesting liquid biopsy as a promising source of material for genetic diagnosis [21,22]. However, liquid biopsy for tumour biopsy genetic analysis can be influenced by TME [23], possibly resulting in an underestimated number of cases of non-detected ITH, and aberrations driving tumour progression or therapy resistance could therefore remain hidden [24].

ITH of both MNA (hetMNA) and typical deletion at 1p36 of the NB have been detected by fluorescence in situ hybridisation (FISH) in different studies [5,25], one of which reports hetMNA as the apparent culprit of aggressive local growth and development of metastases in patients ≥18 months of age at diagnosis [5]. Moreover, ITH has also been detected in other large SCAs, as well as in single nucleotide variations (SNVs), such as those of the *ALK* gene [21,22,26,27]. ITH is commonly associated with progressive disease and treatment resistance, likely because of selection of treatment-resistant clones [2]. However, whether intra-tumoral diversity data can be used to predict clinical outcome has until now remained unclear [28].

In this paper we document an in-depth study of spatial ITH, including several pieces of *MYCN* non-amplified (non-MNA), homogeneously amplified (MNA), and hetMNA primary HR-NB tumours. We applied pangenomic techniques to detect chromosomal aberrations in multiple areas of each tumour and/or in ctDNA from liquid biopsies. Our results highlight the need to analyse at least two solid tumour areas and liquid samples to gain an overview of the entire chromosome landscape at a particular time point, which provides more accurate genomic diagnosis, prognosis and therapy options.

## 2. Materials and Methods

### 2.1. Patients and Samples

A total of 129 samples from 58 NB patients diagnosed between 2002 and 2018 were included in this study, all classified as HR-NB following INRG criteria [8]. Diagnostic biopsy analysis found 32 cases of non-MNA, 20 of MNA and six of hetMNA. We analysed 93 DNA samples from frozen or formalin-fixed paraffin-embedded (FFPE) fragments and 36 ctDNA samples from liquid biopsy (peripheral blood or bone marrow) (Table 1). Given the aim of this work to study spatial ITH, liquid biopsy samples were collected at the same time as the corresponding primary tumours to avoid temporal ITH.

All samples were sent to the Spanish Reference Centre for NB Molecular and Pathological studies (Department of Pathology, University of Valencia-INCLIVA). Histopathological data were provided by the group pathologist. Clinical data were provided by the attending paediatric oncologist where possible, or by the Reference Centre for NB Clinical Studies, including outcome data on event free survival (EFS), defined as length of time from date of diagnosis to any progression, death or to the date of last contact; and overall survival (OS), defined as length of time from date of diagnosis until death or last medical check-up in surviving patients.

### 2.2. MYCN, 1p and 11q Status Detected by FISH

FISH analyses for the *MYCN* gene were performed on tumour imprints and/or FFPE sections of the tumour with *MYCN* (2p24)/*AFF3* (2q11) (KreatechTM, Amsterdam, Netherlands) probe and 4,6-diamidino-2-phenylindole (DAPI) probe as a counterstain. Definition of MNA versus hetMNA samples was as previously described [5]: all malignant neuroblasts of the sample with MNA (some *MYCN* gain also allowed), versus some with MNA and others with whole or numerical copy number anomalies of chromosome 2. FISH of 1p and 11q regions were also performed in most samples, with the *SRD* (1p36)/SE1 and *KMT2A*/SE11 (KreatechTM, Amsterdam, Netherlands) probes, respectively. FISH results were completed with pangenomic techniques.

### 2.3. DNA and ctDNA Extraction

DNA from 83 frozen and 10 FFPE tumours was extracted as described elsewhere [29]. A ctDNA study was carried out on 36 plasma samples extracted with QIAamp Circulating Nucleic Acid (Qiagen, Manchester, UK) kit, following the provided protocol.

### 2.4. SNPa and HD-SNPa Pangenomic Analysis of Tumour Samples

The vast majority of tumour samples (112) were analysed using different high density single nucleotide polymorphism arrays (HD-SNPa): Affymetrix kits used were CytoScan HD DNA for frozen tumours, and OncoScan CNV gene chips for DNA from FFPE tumors and for ctDNA from plasma samples, following by analyses using Chromosome Analysis Suite 3.2 (ChAS) software (ThermoFisher Scientific, Waltham, MA, USA) and Nexus 10.0 Copy Number Discovery (BioDiscovery, El Segundo, CA, USA). The Illumina kits (California, USA) used were HumanCytoSNP-12 for frozen tumour DNA samples and HumanCytoSNP FFPE-12 for FFPE ones, following analysis with GenomeStudio and KaryoStudio software, version 1.4. Only 12 tumour DNA samples were analysed by SNPa with GeneChip Human Mapping 250K (ThermoFisher Scientific, Waltham, MA, USA). Primary data analysis was performed using GDAS 3.0.2 software (ThermoFisher Scientific, Waltham, MA, USA), while genomic profiles were generated using CNAG (Copy Number Analyzer for Affymetrix GeneChip Mapping arrays) version 3.0, with the AsCNAR function (allele-specific copy-number analysis using anonymous references). DNA amplification, tagging and hybridization to gene chips were performed according to the manufacturer’s protocol.

### 2.5. MLPA Analysis of Tumour Samples

Only five tumour DNA samples belonging to five patients were analysed with this technique and were compared to HD-SNPa results to study ITH. MLPA was performed using the SALSA MLPA Kit P251/P252/P253 developed by MRC-Holland in cooperation with the International Society of Paediatric Oncology European Neuroblastoma (SIOPEN). The technique and interpretation guidelines are as described elsewhere [30].

### 2.6. ALK Mutations and ALT Status

*ALK* mutations were studied in 37 out of 58 cases, using Sanger, next generation sequencing (mean depth 80×), or targeted deep sequencing (TDS) approaches. All detected mutations were validated by a second independent experiment [31]. Alternative lengthening of telomeres (ALT) were analysed using c-circle assay [32] in 17 of 58 tumours, all non-MNA.

### 2.7. Statistical Analysis

All data were analysed using SPSS analysis software (version 26). Pearson’s χ2 statistic was used to identify differences in frequency of each genomic aberration between HR-NB groups. *p*-values under 0.05 were considered statistically significant. Survival analyses were carried out using Kaplan-Meier curves. Additionally, multivariate Cox regression analysis was undertaken with stepwise, forward and backward (Wald) methods to calculate the impact of ITH on patient survival.

## 3. Results

### 3.1. Combined Samples HD-SNPa Revealed High ITH

**Figure 1:**
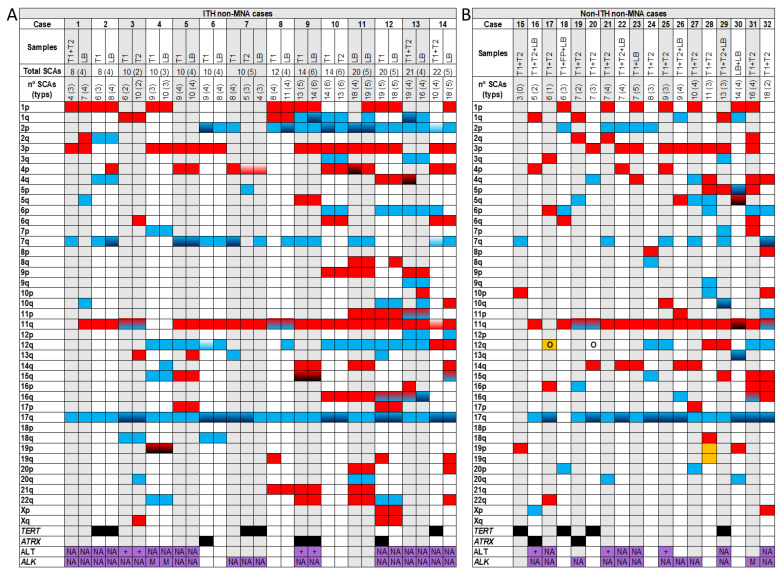
Genomic aberrations found by SNPa in *MYCN* non amplified cases (non-MNA) with intra-tumour heterogeneity (ITH) (**A**) and non-ITH (**B**). In the upper part of both Figures A and B the analysed samples (T: solid tumour -1, 2-; LB: liquid biopsy) corresponding to each case (1–32) with the total number of segmental chromosomal aberrations (SCAs) and the typical ones in parenthesis, are shown. In Figure 1A the total number of SCAs and typical ones of each case, taking into account all the analysed samples, are also presented. Solid blue 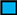 or red 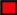 squares refers to gains or losses, respectively in the indicated chromosome arm. Gradient color from blue 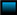 or red 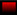 to black indicates gains or losses with two or more chromosome fragments in the chromosome arm. Gradient color from white to blue 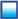 or red 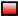 indicates changes in the break position (which results in size variations of the aberration) in the chromosome arm between the analyzed samples of a case (only in ITH ones). Gradient color from blue to red 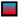 refers to both gains and losses in the same chromosome arm of a sample. Solid yellow 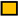 squares indicate the presence of a chromotripsis-like phenomenon. Black squares 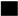 indicate the presence of focal segmental chromosomal aberrations (FSCAs) affecting *TERT* or *ATRX* genes. Purple squares 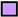 indicate that the samples have been analysed for alternative lengthening of telomeres (ALT) or for mutations and amplifications in the *ALK* gene; NA: in the squares indicates that any aberration has been detected in the sample; +: refers to the presence of ALT; A: refers to presence of *ALK* amplification; M: indicates ALK mutation. O: indicates amplification of any gene or region in the chromosome arm, excluding *MYCN* or *ALK* amplification.

**Figure 2:**
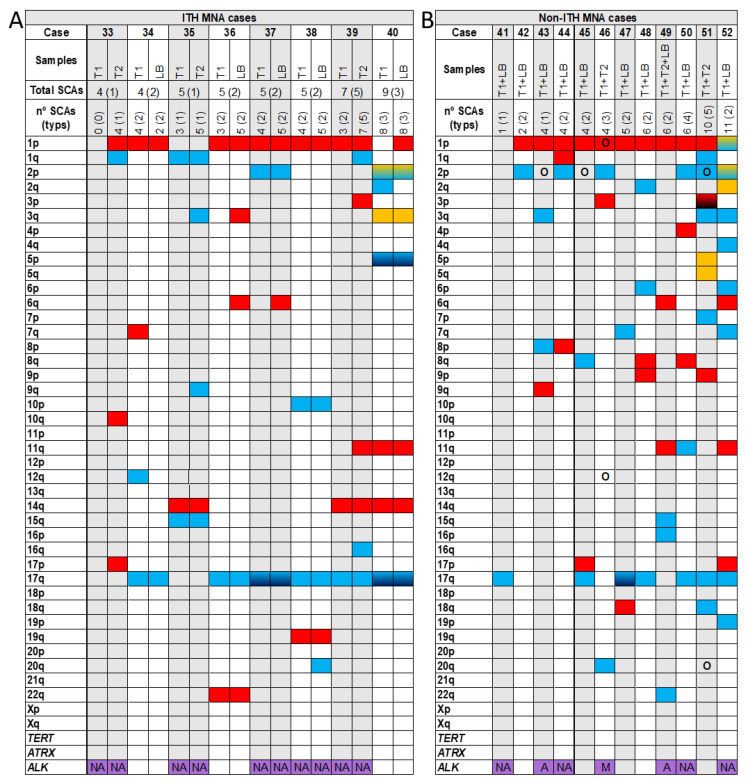
Genomic aberrations found by SNPa in *MYCN* amplified cases (MNA) with intra-tumour heterogeneity (ITH) (**A**) and non-ITH (**B**). In the upper part of both Figures A and B the analysed samples (T: solid tumour -1,2-; LB: liquid biopsy) corresponding to each case (33–52) with the total number of segmental chromosomal aberrations (SCAs) and the typical ones in parenthesis, are shown. In Figure 2A the total number of SCAs and typical ones of each case, taking into account all the analysed samples, are also presented. Solid blue 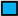 or red 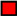 squares refers to gains or losses, respectively, in the indicated chromosome arm. Gradient color from blue 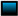 or red 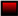 to black indicates gains or losses with two or more chromosome fragments in the chromosome arm. Solid yellow 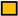 squares indicate the presence of a chromotripsis-like phenomenon, and gradient color from yellow to blue 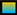 refers to chromotripsis-like phenomenon within a gain. Purple 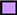 squares indicate that the samples have been analysed for mutations and amplifications in *ALK* gene; NA: in the squares indicates that any aberration has been detected in the sample; A: referes to presence of *ALK* amplification; M: indicates *ALK* mutation. O: indicates amplification of any gene or region in the chromosome arm, excluding *MYCN* or *ALK* amplification.

**Figure 3:**
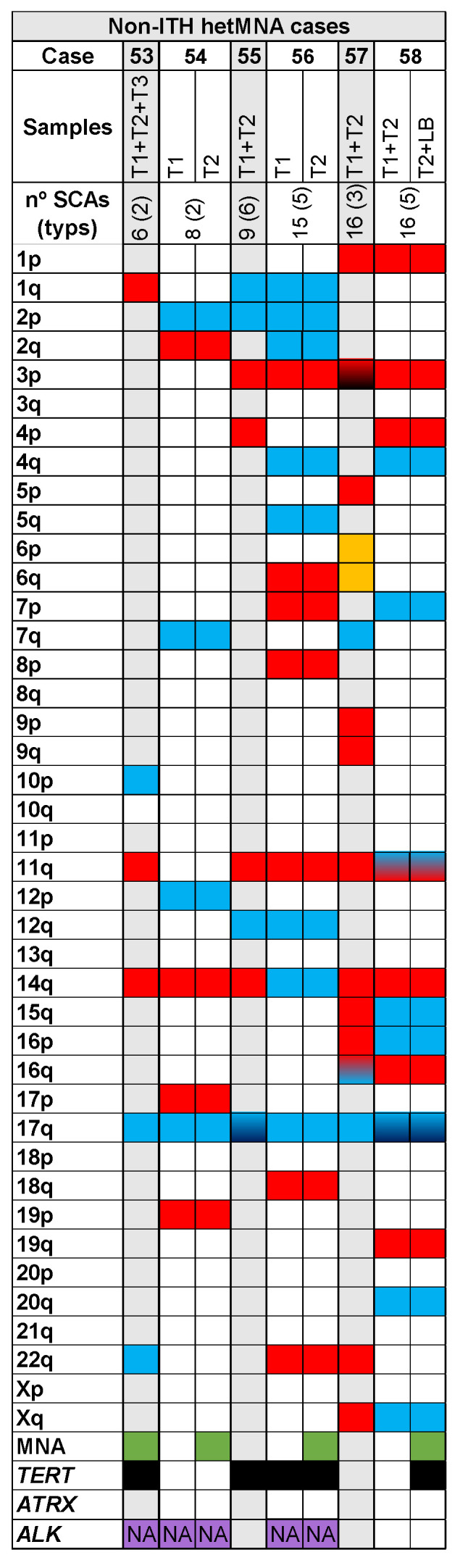
Genomic aberrations found by SNPa in cases with heterogeneous *MYCN* amplification (hetMNA). In the upper part the number of the analysed samples (T: solid tumour -1, 2, 3-; LB: liquid biopsy) corresponding to each case (53–58) with the total number of SCAs and the typical ones in parenthesis, are shown. Solid blue 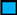 or red 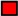 squares refers to gains or losses, respectively in the indicated chromosome arm. Gradient color from blue 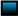 or red 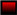 to black indicates gains or losses with 2 or more chromosome fragments in the chromosome arm. Gradient color from blue to red 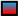 refers to both gains and losses in the same chromosome arm of a sample. Solid yellow squares 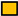 indicate presence of chromotripsis-like phenomenon. Green squares 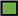 indicate *MYCN* amplification detection by SNPa. Black squares 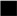 indicate presence of focal segmental chromosomal aberrations (FSCAs) affecting *TERT* or *ATRX* genes. Purple squares 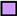 indicate that the samples have been analysed for alternative lengthening of telomeres (ALT) or for mutations and amplifications in *ALK* gene; NA: in the squares indicates that any aberration has been detected in the sample.

Among the 58 HR-NB cases included in our study, 22 cases (37.9%) had ITH from the SCAs observed in pangenomic analysis between patient samples, from either tumour or liquid biopsy (Table 1 and Figure 1, Figure 2 and Figure 3). Specifically, we found ITH by SNPa in 14 out of 32 non-MNA cases (44%), in eight out of 20 MNA HR-NB cases (40%) but not in either of the hetMNA cases (Table 1).

Different solid areas of the same tumour were analysed in 24 out of 58 cases (49 out of 93 studied DNA samples), and five of them (21%) showed ITH: 13 tumours were non-MNA, of which only two had ITH (15%), six were MNA and three of them presented ITH (50%) and the remaining five were hetMNA, in which HD-SNPa found no differences in SCAs (Table 1). We analysed only liquid biopsies (from peripheral blood or bone marrow aspirate) in two non-MNA tumours, one of which had ITH (Figure 1A,B). We detected ITH in 16 (50%) of the 32 cases in which one or more solid plus liquid biopsies were analysed (in total, 44 out of 93 analysed DNA samples from different tumour areas, plus 32 out of 36 studied ctDNA samples). Among the 32 cases with both type of samples analysed, 17 were non-MNA, showing ITH in 11 cases (65%); 14 were MNA, five of which presented with ITH (36%), and one was an hetMNA case without ITH (Table 1). Among the 16 cases with ITH, in 10 we detected more SCAs in liquid than in solid samples, while in five cases the opposite was true. The remaining case showed an equal number of SCAs, but with between-sample differences in some SCAs (Figure 2A, case 40).

Of the 6 hetMNA cases (Figure 3), none showed ITH as reflected in SCAs from the different samples analysed by HD-SNPa. However, FISH of those tumours showed heterogeneity in *MYCN* amplification and also in loss at 1p (Figure 3, cases 53 and 54) or loss at 11q (Figure 3, case 58).

The number of chromosomal aberrations in ITH cases ranged from 4 to 23, with an average of 3.55 genomic events which differed between ITH tumour samples (an average of 36% differing genomic events) (Table 2). Besides differential presence/absence of SCAs, we also observed heterogeneous cell clones with shifts in the chromosome break position in 3 tumours, and presence of additional chromosome breaks in broken chromosomal regions in 5 cases, apparently resulting in altered SCA size. In addition, we detected ITH of FSCAs (focal SCAs < 3Mb) in *TERT* (2 non-MNA cases) and *ATRX* genes (2 non-MNA cases). Presence of any of these genomic aberrations was considered a genomic event for statistical analysis. Finally, only one genomic event, gain at 12q, was significantly positively associated with ITH (*p*-value = 0.014), but this event was not associated with survival.

### 3.2. High Average Number of SCAs in ITH Non-MNA Samples

All the studied tumours presented SCA profiles, with an average of 9.2 and a median of 8 SCAs. However, we observed a significant difference (*p*-value < 0.01) in the number of SCAs between tumour types related *MYCN* status when we distributed data into quartiles, with ITH non-MNA tumours having more SCAs (average 13.3, Figure 1A) than non-ITH non-MNA ones (average 8.8, Figure 1B). Within MNA cases we found similar average SCAs in ITH and non-ITH cases (5.5 and 5.3, respectively, Figure 2A,B). Finally, hetMNA had an average of 11.7 SCAs (similar to non-MNA, Figure 3).

**Figure 4:**
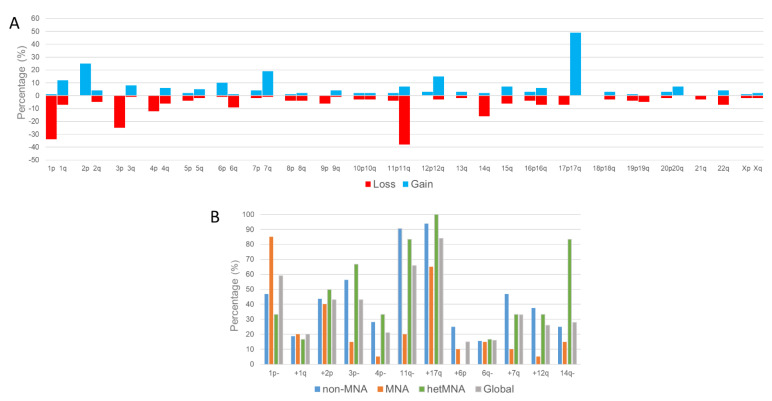
Percentage of the analysed cases that showed (**A**) segmental chromosomal aberrations in each chromosome arm (losses of genomic material are in red, and gains in blue). (**B**) Percentage of each segmental chromosome aberration observed in the cases MYCN non-amplified (non-MNA, blue), MYCN amplified (MNA, orange) or with heterogeneous amplification of MYCN (hetMNA, green), and in all the analyzed high-risk neuroblastoma cases (grey).

Across the whole cohort, in addition to the high number of the typical SCAs, we found considerable incidence of certain atypical SCAs: +6p, 6q−, +7q, +12q and 14q− (Figure 4A). Some SCAs showed significant differences (*p* < 0.05) in frequency between non-MNA, MNA and hetMNA tumours. Most notable among the typical SCAs were losses at 3p and 4p, while gains at 7q and 12q were predominant among the atypical ones with more prevalence in non-MNA and hetMNA cases. As is already known, 11q− is also less common in MNA tumours (Figure 4B). Curiously, 14q− was present in 5 (83%) of the hetMNA cases; this SCA was only present in a few tumours of the remaining groups.

Furthermore, large chromosome segments or whole chromosome copy number loss of heterozygosity (cnLOH) was present in 18 (31%) cases. A chromotripsis-like phenomenon involving eight chromosomes was detected in 6 cases (2 non-MNA, 3 MNA and 1 hetMNA) without ITH being observed in this genomic event. FSCAs affected 11 cases (18%) of *TERT*, showing ITH in 2 of them. *ATRX* FSCAs were detected in 5 cases (9%), 2 of which displayed ITH. No FSCAs of either gene were detected in MNA cases. *ALK* mutation was found in 3 of 37 studied tumours (2 MNA and 1 non-MNA) and amplification in 2 out of 58 cases (both MNA). Finally, ALT was present in 5 out of 17 analysed cases (Figure 1, Figure 2 and Figure 3).

### 3.3. Improved ITH Detection in Non-MNA Samples with Combined Solid and Liquid Biopsy Analysis

Of the 32 non-MNA primary tumours, 14 showed spatial ITH (43.75%), found in 65% of non-MNA tumours when at least one solid and one liquid biopsy were analysed (11 out of 17), and 15% of cases with two or more solid areas analysed (2 out of 13). Total number of SCAs was higher in solid tumour DNA than in ctDNA in 4 tumours, with the opposite in 7. However, in some cases SCAs were only detectable in solid samples, and others only in liquid biopsy (Figure 1A). One patient (Figure 1A, case 7) showed differences between two tumour areas and in liquid biopsy. In the last ITH non-MNA case, liquid biopsy varied between bone marrow and peripheral blood samples (1 out of 2, Figure 1A, case 11).

TypSCAs and some atypSCAs were found in greater proportion in ITH than non-ITH cases (Table 3). Moreover, differences in genomic events between samples from ITH non-MNA tumours ranged from 1 to 14, with ITH found in 7–73% of events (average 31%). The most frequent ITH genomic events were +7q and +2p (in 5 cases). 4 cases also had ITH in TERT (cases 7 and 14) or ATRXs (cases 6 and 12) genes (Table 2, Figure 1A,B).

### 3.4. ITH Detected in 40% of MNA Cases

Of 20 MNA cases, 8 showed between-sample differences in pangenomic profile (Figure 2A,B). Specifically, we found SCA differences in 3 out of 6 of analysed cases with at least two solid samples (50%), and in 5 of 14 with solid and liquid samples (36%). One case (Figure 2A, patient 33) had no SCAs in one tumour sample and 4 SCAs in another. One case had more SCAs in tumour DNA than in ctDNA (Figure 2A, case 34), another case had an equal number of SCAs but some were different between tumour and liquid biopsy (Figure 2A, case 40) and 3 cases showed more SCAs in liquid biopsies than in solid ones.

Small differences between ITH and non-ITH cases were found in the number of SCAs: an average of 5.5 (median 5) vs. 5.3 (median 4.5), respectively, and in the type of typ- and atypSCAs (Table 3). The number of between-sample differences in genomic events ranged between 1 and 4 SCAs. ITH was found in 20–57% of events (average, 36%), a similar proportion to that observed in non-MNA cases (31%). No SCAs with especially high ITH between tumour biopsies were detected.

### 3.5. Influence of ITH on EFS and OS

**Figure 5:**
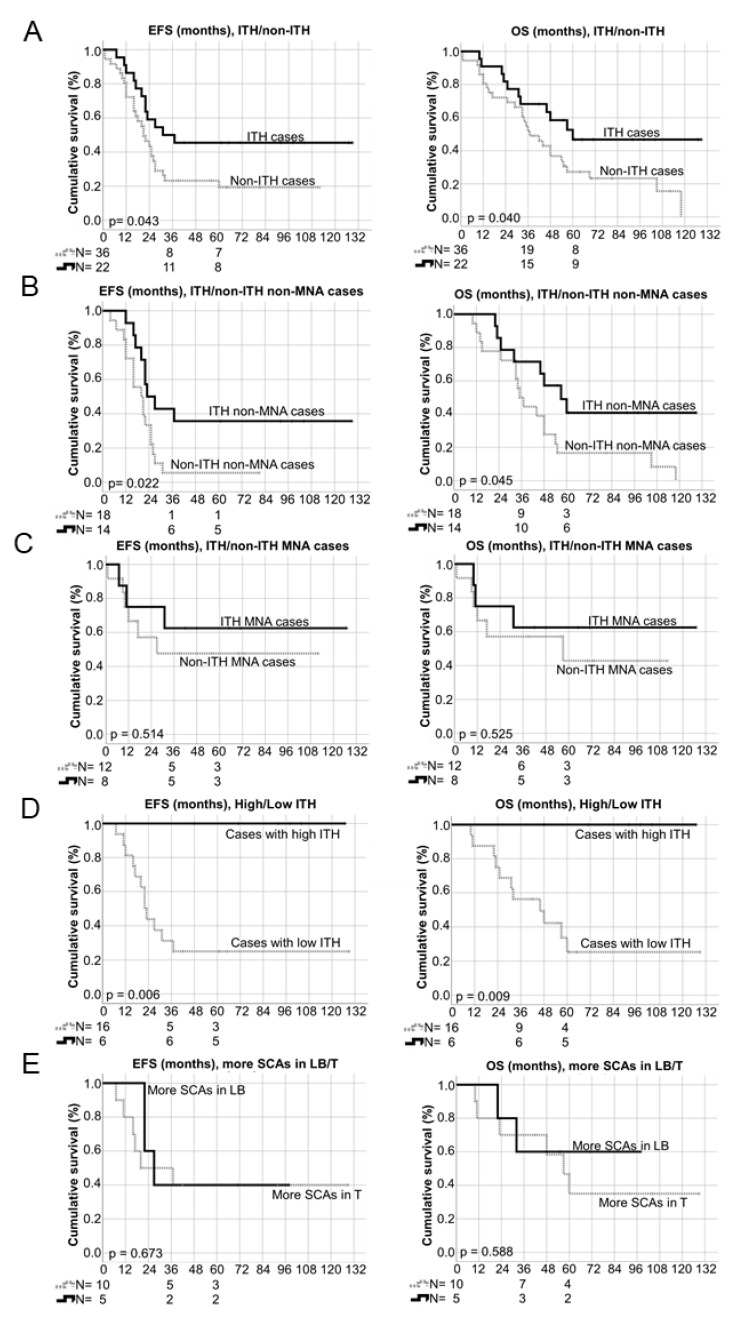
Kaplan–Meier graphs showing event free and overall survivals (left side, EFS and right side, OS) depending on different variables: (**A**) intra-tumour heterogeneity (ITH) detected or not detected (non-ITH) in all the studied cases; (**B**) ITH detected or not detected (non-ITH) in MYCN non-amplified (non-MNA) cases; (**C**) ITH detected or not detected (non-ITH) in MYCN amplified (MNA) cases; (**D**) high ITH (tumors with 50% or more different genomic events between the analysed samples) versus low ITH (tumours with ITH but with less than 50% different genomic events between the analysed samples); (**E**) higher number of segmental chromosomal aberrations (SCAs) observed in liquid biopsy (LB) versus higher number in solid tumour (T) samples. N: number of cases. 3-year and 5-year number of survivors, as well as *p*-values are shown.

Event free and overall survivals were followed up from 1 to 129 months (average 36.5 and 47.7 months, respectively). Notably, longer survival was observed at 36 and 60 months (EFS *p*-value = 0.043, OS *p*-value = 0.041, Figure 5A) in patients with ITH than in non-ITH patients. This survival difference was still significant taking only non-MNA cases, which showed a 43% survival rate in ITH cases compared with only 15% in non-ITH tumours (Figure 5B). However, survival was not statistically associated with ITH in MNA HR-NB despite a higher rate in ITH cases (62.5%) than in non-ITH (50%) ones (Figure 5C). HetMNA patients showed a 17% survival rate. Following on from this observation, we undertook to study whether differences in prevalence of genomic events between samples from ITH tumours were associated with survival. Surprisingly, we observed 100% survival at 48 months in patients with ITH and at least 50% differences in genomic events, with significant differences in both EFS and OS (*p*-value = 0.009 and 0.006, respectively) (Figure 5D). No significant survival differences were detected in patients with more SCAs in liquid than in solid samples or vice versa (Figure 5E).

Overall, the higher survival in ITH patients was attributable to ITH rather than the other genomic factors that we studied, as was observed in both stepwise forward and backward (Wald) Cox regression analysis. However, EFS cox regression test (backward, Wald) also showed as significant *MYCN* status. In addition, we investigated the association with survival of number of SCAs, typSCAs and the most frequently found atypSCAs, presence of cnLOH and FSCAs in *TERT* and *ATRX* genes, none of which showed significant *p*-values (*p* < 0.05, Table 4).

## 4. Discussion

An aberrant TME can promote genetic instability and could even compromise the DNA repair pathways needed to prevent malignant transformation [3,33,34]. Tumours can have different areas with disparate TME features, which could reflect differing genomic aberrations in tumour cells. It is well-known that the centre of the tumour typically has fewer blood vessels and more hypoxia than tumour borders [35], which can influence pH and metabolism, and also induce genomic and epigenomic changes between zones [36]. However, tissue areas of tumours submitted to genetic diagnostics yield little information even without taking ITH into account and this could be among the principal reasons for low reproducibility, not only in diagnostics and treatment but also in bio-medical research [1,37].

The INRG Biology Committee has already recommended ensuring that a sufficient amount of tumour material is analysed, as determined by a pathologist, from at least two different tumour regions in NB genetic diagnosis [38]. Guidelines have also been published on optimal tumour section for detecting genomic ITH [39]. However, it should be noted that NB are frequently found in neonates and young children, in whom total surgical excision or representative biopsy collection are restricted options [40]. These difficulties in NB tumour sampling result in underdetection of genomic aberrations located in metastatic clones, forming small foci, nodules (such as ganglioneuroblastoma, nodular), or dispersed in an isolated tumour area that could lead to erroneous genetic diagnosis and inadequate treatment selection [41,42].

In our study we detected genetic spatial genomic ITH with HD-SNPa in 21% of cases with at least two tumour pieces analysed, which permitted a greater representation of overall TME involvement. This considerable percentage shows the importance of analysing the largest possible area of the tumour, which can be achieved by mixing material from different tumour areas to obtain DNA from diverse TME locations. In 50% of cases ITH detection was enhanced, when we obtained DNA from one or more tumour specimens and ctDNA from liquid biopsy. These results demonstrate not only the extent of tumour heterogeneity, but also the sensitivity of liquid biopsy in SCA detection, in agreement with reports over recent years [21,22,43,44,45]. Cases in which more genomic aberrations were detected in liquid biopsy than in solid tumour may reveal not only different clones of the biopsied tumour, but also that several clones may have invaded other parts of the body, or were trying to do so [46]. Nevertheless, in some cases we detected fewer genomic aberrations in liquid biopsy than solid tumour. Presence of genomic aberrations in the former biopsy could also be conditioned by the TME. The proportion of necrosis, variations in non-tumour cells, as well as permeable blood and lymphatic vessels, among others, determines the different amount of ctDNA in the liquid biopsy released by different tumour areas [47]. In addition, a heterogeneous TME may render liquid biopsy results incompletely representative of the entire tumour. Consequently, liquid biopsy should be a complementary source of tumour DNA, but not a substitute for the whole biopsy [48,49], and should only become an alternative in cases in which tumour tissue biopsy is precluded.

A recent study comparing genetic results derived from solid tumours and liquid samples of NB suggested that solid tumour-specific alterations could correspond to clones that might release less ctDNA, meaning less aggressive cells, while ctDNA-specific alterations could correspond to more aggressive clones or to those originating from metastatic sites [22]. We found no significant results comparing patient survival rates.

Interestingly, patients with ITH had better EFS and OS when we included all the studied cases in the Kaplan-Meier analysis, results that were replicated when patients with non-MNA tumours were analysed separately, but in contrast were not statistically significant for MNA cases, despite their higher rate of survival in ITH ones. Taking into account these results, we expanded our ITH analysis to study whether the number of between-sample differences in genomic events in the same patient was associated with survival. When at least 50% of genomic events differed between samples, indicating with high ITH tumours, all patients were found to survive.

Various hypotheses could explain this finding. Patients with greater ITH could survive due to the competition between several clones, possibly influenced by a heterogeneous TME. This has been described as occurring most typically during early tumour formation and proliferation, when tumour cells are trying to adapt to the niche, in contrast to non-heterogeneous tumours in which an aggressive and predominant clone has already become established, conferring worse prognosis [1,3]. Among cell populations trying to respond to adverse TME features, only the most pheno-genotypically adapted ones will succeed in invading tissues [50,51]. The most advantageous tumour cells will also segregate anchor proteins and transform the ECM into one with favourable biochemistry and biotensegrity, which will also promote homogeneous establishment of the powerful clones, allowing them to grow and migrate [20,33]. Another explanation could be that ITH cases, especially non-MNA ones, have high genomic instability as reflected in a high number of SCA cell profiles, which they cannot withstand and thus collapse earlier than tumours with lower SCA numbers. Both genomic instability and degree of tumour ITH have previously been associated with poor prognosis, conferring on the tumour more opportunities to adapt and resist chemotherapy [52,53,54]. However, extreme levels of genomic instability have been reported to be associated with increased OS [52] and patients with tumours displaying more than four different sub-clones have reduced mortality risk [55]. It has been observed that cancer cells with these levels of genomic instability rarely replicate successfully, and those tumours may also be more immunogenic [47], suggesting a trade-off between the costs and benefits of genomic instability [55]. This is exemplified by the phenomenon of chromotripsis as a chaotic genomic event. It has been shown that genomic chaos leads to reorganization that could persist over time, but also that cells with too much genomic instability are usually eliminated during cancer development [56]. A recent study published by our group in a MNA NB cell line grown in stiff TME models observed disappearance of clones with high SCAs and chromotripsis-like phenomena and positive selection of clones with specific SCAs affecting mechanosignalling genes [3].

Our findings support a revised approach to the currently reported frequency of several recurrent SCAs and their involvement in HR-NB. We detected greater incidence of certain atypical SCAs (such as gains at 7q, 12q, and loss at 14q) than of other typical SCAs like 1q gain or 4p loss. Moreover, some had differing prevalence in non-MNA, MNA and hetMNA tumours, and these associations should be taken into account. Gain at 7q was mainly found in non-MNA and hetMNA cases, and was differentially found across tumour samples. Various publications have also described a high presence of this aberration in NB, especially in stage four [57,58]. Although gain at 12q is understudied in the scientific literature, amplification (also present in three of the present cases; see Figure 1B and Figure 2B) and overexpression of some of its genes, is correlated with poor prognosis [59,60]. In our cohort, gain of this region was most present in ITH cases, and preferentially in non-MNA and hetMNA tumours. Deletion and loss of heterozygosity of 14q has been also previously reported to be involved in NB initiation and progression [61,62,63]. In the cohort studied herein, these alterations were most frequent in hetMNA cases. Finally, we also detected high frequency of FSCAs in *TERT* and *ARTX* genes, which are involved in telomere maintenance through telomerase activity and alternative lengthening of telomeres [64]. Both genes are considered important in understanding the etiology and molecular pathogenesis of NB, and hence for identifying diagnostic and treatment markers. We found FSCAs in these genes differentially present among some analysed HR-NB samples. As development of telomerase inhibitors and identification of alternative lengthening of telomere-related targets becomes a reality [64], detection of both gene aberrations, even in ITH, by analysing several tumour samples will play a key role.

We detected ITH in almost 40% of our samples, and the true percentage could even be higher. The HD-SNPa obtained from each tumour sample reflects the chromosomic aberrations of an aggregate of minor clones that coexist in that piece, a metagenome. However, HD-SNPa software analysis leads to easy ITH detection with at least 30% allelic frequency, or even at lower proportions in good quality samples [3,65], although very uncommon aberrations are difficult to detect. This would be the case in tumours without observed between-sample ITH, which could have small-scale heterogeneity, thus conferring poor prognosis. It has been reported that small sub-clones are usually associated with poor prognosis, as they can adapt but later evolve into tumour progression, or also lead to new sub-clones [55]. Moreover, we investigated SCAs as the most frequently found genetic aberrations in NB, and a great source of genomic instability; however, there are a wealth of alternative genetic, epigenetic and transcriptomic aberrations which could also confer ITH [22,66,67]. These data are especially pivotal in the current panorama in which identification of several well-known and other less explored genetic aberrations are essential for therapeutic stratification, as tools in patient clinical guidance and as potential treatment targets (typical SCA, *ALK* mutation, ALT alterations). Moreover, beyond its importance in diagnosis, prognosis and therapy, ITH detection can also provide greater insight into the internal composition of tumours [1]. In-depth research with powerful new genomic approaches, combined with digital pathology analysis of the TME may be key to understanding the molecular and cellular underpinnings of NB and other cancers. In addition, the association between ITH and improved prognosis in the cohort studied suggest that efforts aimed at modulating the cost/benefit trade-off of unstable tumours may represent a new therapeutic avenue [55].

## 5. Conclusions

As ITH is associated with survival in our HR-NB patients, and especially in non-MNA tumours, further studies with larger NB cohorts are clearly warranted to investigate its value as an independent prognosis factor. If ITH really implies increased survival, then this should be taken into consideration and review and possible adjustment of standard therapy should be encouraged. A more detailed study of ITH, potentially revealing genomic aberrations present in only a few cells, will also help decision-making on targeted treatment. Analysing multiple tumour samples (a mix of their DNAs) plus liquid biopsy to make a routine accurate genomic diagnosis seems an optimal approach to detect ITH and move towards improved precision medicine. Whole genome (or exome) sequencing, digital PCR and single-cell RNA-Seq are powerful techniques to detect and confirm genomic ITH, not only in SCAs but also in single nucleotide variants. Combined with digital image analysis of the TME, these genomic techniques will be useful to detect the ITH of the whole tumour, at genomic and morphological/topological levels, to predict the course of tumour clones and ultimately to determine prognosis and the best treatment options for each patient.

## Figures and Tables

**Table 1:** Intratumor heterogeneous (ITH) cases according to total high-risk neuroblastomas (HR-NB) studied, their genomic profile (MYCN non-amplified, non-MNA; MYCN amplified, MNA; or with heterogeneous amplification of MYCN, hetMNA) and the combination of samples analysed in each case.

ITH Cases/Total HR-NB Cases	ITH Cases/Total Genomic Profile Cases	ITH Cases/Combination of Samples Analysed	
22/58 HR-NB (37.9%)	14/32 non-MNA (44% ITH)	2/13 (15.4%)	T1 + T2
		11/17 (64.7%)	T + LB
		-	7/8 T1 + LB
			4/9 T1 + T2 + LB
		1/2 (50.0%)	LBp + LBm
	8/20 MNA (40% ITH)	3/6 (50.0%)	T1 + T2
		5/14 (35.7%)	T + LB
		-	5/13 T1 + LB
		-	0/1 T1 + T2 + LB
	0/6 hetMNA (0% ITH)	0/5 (0.0%)	T1 + T2
			0/4 T1 + T2
			0/1 T1 + T2 + T3
		0/1 (0.0%)	T + LB
			0/1 T1 + T2 + T3+ LB
-	-	**Total**	93 T
-	-		36 LB
-	-		129 samples

T: solid tumor (1, 2, 3); LB: liquid biopsy, LBp: liquid biopsy from peripheral blood, LBm: liquid biopsy from medullary aspirate.

**Table 2:** Genomic events and survival data in intra-tumour heterogeneous (ITH) cases. Cases with high ITH (with 50% or more of genomic events different between the analysed samples) are highlighted.

Case	Total N Genomic Events	N Diff. Genomic Events between Samples	% Diff. Genomic Events	EFS (Months)	Relapse/Death	OS (Months)	Death
1	8	5	62.50	92	No	92	No
2	9	2	22.22	37	Yes	60	Yes
3	10	4	40.00	23	Yes	46	Yes
4	10	1	10.00	16	Yes	48	Yes
5	10	1	10.00	129	No	129	No
6	11	3	27.27	27	Yes	55	No
7	11	8	72.73	98	No	98	No
8	12	5	41.67	20	Yes	23	Yes
9	15	1	6.67	17	Yes	57	Yes
10	14	1	7.14	12	Yes	25	Yes
11	20	4	20.00	61	No	61	No
12	21	4	19.05	22	Yes	22	Yes
13	21	7	33.33	22	Yes	32	Yes
14	23	14	60.87	104	No	104	No
33	4	4	100.00	127	No	127	No
34	4	2	50.00	71	No	71	No
35	5	2	40.00	31	Yes	31	Yes
36	5	2	40.00	42	No	42	No
37	5	1	20.00	7	Yes	10	Yes
38	5	1	20.00	11	Yes	11	Yes
39	7	4	57.14	48	No	48	No
40	11	2	18.18	65	No	65	No
Average	10.95	3.55	35.40	49.18	-	57.14	-
Median	10.00	2.50	30.30	34.00	-	51.50	-

**Table 3:** Percentage of the analysed cases with or without intratumor heterogeneity (ITH, and non-ITH, respectively) and without or with *MYCN* amplification (non-MNA and MNA, respectively) that showed typical segmental chromosomal aberrations (SCAs), the most frequently found atypical SCAs, and other genomic aberrations as focal SCAs in *TERT* and *ATRX* genes, number of cases with presence of alternative lengthening of telomeres (ALT positive), and presence of mutations (mut) or amplification (amp) in *ALK* gene.

Cases	Typical SCAs							Atypical SCAs								Other Aberrations			
	1p−	+1q	+2p	3p−	4p−	11p−	+17q	+6p	6q−	+7q	9p−	+12q	14q−	19p−	21q−	*TERT*	*ATRX*	ALT	*ALK*
ITH non-MNA	57%	29%	64%	64%	43%	93%	100%	28%	21%	64%	21%	57%	21%	21%	21%	21%	21%	2 positive	1 mut, 0 amp
non-ITH non-MNA	39%	11%	28%	50%	17%	89%	89%	22%	11%	33%	0%	22%	27%	0%	0%	22%	11%	3 positive	1 mut, 0 amp
ITH MNA	87%	38%	25%	13%	0%	25%	75%	0%	25%	0%	0%	13%	38%	0%	0%	0%	0%	-	0 mut, 0 amp
non-ITH MNA	83%	8%	50%	17%	8%	17%	58%	8%	8%	16%	16%	0%	0%	0%	0%	0%	0%	-	1 mut, 2 amp

**Table 4:** Cox regression tests results after applying stepwise forward and backward Wald methods. MYCN status (MYCN non-amplified (non-MNA), MYCN amplified (MNA) heterogeneous amplification of MYCN (hetMNA)) intra-tumour heterogeneity (ITH) and the segmental chromosomal aberrations 3p−, +12q and 14q− are the variables that are maintained in the last step after applying the Wald backward stepwise method, according to event-free survival (EFS) in the studied high-risk neuroblastoma patients, being ITH and MYCN status the only significant ones (*p*-value < 0.05). In the rest of the applied methods ITH is the only maintained variant. B: Beta coefficient; SE: Standard Error; CI: Confidence interval. Other variables without significant results included in the Cox regression analysis were: number of segmental chromosomal aberrations (SCAs) by quartiles, copy number loss of heterozygosity, aberrations in TERT and ATRX, typical SCAs (1p−, +1q, +2p, 4p−, 11q−, +17q) and the atypical SCAs (+6p, 6q−, +7q).

Variable	B	SE	Wald	Exp(B) (95% CI)	Sig.
**EFS Wald (forward stepwise) method**					
ITH	−0.677	0.347	3.811	0.508 (0.257–1.003)	0.051
**EFS Wald (backward stepwise) method**					
ITH	−1.170	0.397	8.694	0.310 (0.143–0.675)	0.003
MYCN status	−0.628	0.278	5.102	0.534 (0.310–0.920)	0.024
3p-	−0.690	0.405	2.901	0.502 (0.227–1.110)	0.089
+12q	0.702	0.423	2.759	2.019 (0.881–4.624)	0.097
14q-	0.816	0.438	3.469	2.262 (0.958–5.339)	0.063
**OS Wald (forward stepwise) method**					
ITH	0.716	0.358	4.000	0.489 (0.242–0.986)	0.046
**OS Wald (backward stepwise) method**					
ITH	0.716	0.358	4.000	0.489 (0.242–0.986)	0.046

## Data Availability

No new data were created or analyzed in this study. Data sharing is not applicable to this article.
